# Predictive ability of visit-to-visit glucose variability on diabetes complications

**DOI:** 10.1186/s12911-025-02964-2

**Published:** 2025-03-17

**Authors:** Xin Rou Teh, Panu Looareesuwan, Oraluck Pattanaprateep, Anuchate Pattanateepapon, John Attia, Ammarin Thakkinstian

**Affiliations:** 1 Department of Clinical Epidemiology and Biostatistics, Faculty of Medicine Ramathibodi Hospital, Mahidol University, 270 Rama VI Road, Phaya Thai, Bangkok, 10400 Thailand; 2 Centre for Clinical Outcome Research, Institute for Clinical Research, National Institutes for Health, Ministry of Health, Shah Alam, Selangor Malaysia; 3 School of Medicine and Public Health, University of Newcastle, Newcastle, NSW Australia

**Keywords:** Glucose variability, Diabetes complications, Coefficient of variation, Prognostic factors, Machine learning

## Abstract

**Background:**

Identification of prognostic factors for diabetes complications are crucial. Glucose variability (GV) and its association with diabetes have been studied extensively but the inclusion of measures of glucose variability (GVs) in prognostic models is largely lacking. This study aims to assess which GVs (i.e., coefficient of variation (CV), standard deviation (SD), and time-varying) are better in predicting diabetic complications, including cardiovascular disease (CVD), diabetic retinopathy (DR), and chronic kidney disease (CKD). The model performance between traditional statistical models (adjusting for covariates) and machine learning (ML) models were compared.

**Methods:**

A retrospective cohort of type 2 diabetes (T2D) patients between 2010 and 2019 in Ramathibodi Hospital was created. Complete case analyses were used. Three GVs using HbA1c and fasting plasma glucose (FPG) were considered including CV, SD, and time-varying. Cox proportional hazard regression, ML random survival forest (RSF) and left-truncated, right-censored (LTRC) survival forest were compared in two different data formats (baseline and longitudinal datasets). Adjusted hazard ratios with 95% confidence intervals were used to report the association between three GVs and diabetes complications. Model performance was evaluated using C-statistics along with feature importance in ML models.

**Results:**

A total of 40,662 T2D patients, mostly female (61.7%), with mean age of 57.2 years were included. After adjusting for covariates, HbA1c-CV, HbA1c-SD, FPG-CV and FPG-SD were all associated with CVD, DR and CKD, whereas time-varying HbA1c and FPG were associated with DR and CKD only. The CPH and RSF for DR (C-indices: 0.748–0.758 and 0.774–0.787) and CKD models (C-indices: 0.734–0.750 and 0.724–0.740) had modestly better performance than CVD models (C-indices: 0.703–0.730 and 0.698–0.727). Based on RSF feature importance, FPG GV measures ranked higher than HbA1c GV, and both GVs were the most important for DR prediction. Both traditional and ML models had similar performance.

**Conclusions:**

We found that GVs based on HbA1c and FPG had comparable performance. Thus, FPG GV may be used as a potential monitoring parameter when HbA1c is unavailable or less accessible.

**Supplementary Information:**

The online version contains supplementary material available at 10.1186/s12911-025-02964-2.

## Background

Diabetes is increasing worldwide with 537 million adults living with diabetes, the majority of them in low- and middle-income countries [1]. This will lead to a further increase in healthcare burden as the cost of illness increases, particularly as patients develop diabetes complications [2]. Identifying diabetes complications early is thus important in diabetes management in order to delay disease progression [3]. Studies that explore the prognostic factors for diabetic complications and develop prognostic models may help to risk-stratify diabetic patients and help clinicians in management and decision-making [4, 5].

Haemoglobin A1c (HbA1c), measures average glycemic control in the past three months, and is a widely used biomarker in diabetic monitoring and prognostic modelling. However, HbA1c reliability will be affected by conditions that are related to red blood cell turnover, such as pregnancy or anaemia [6]. Recently, many studies explored the use of visit-to-visit glucose variability (GV) as an additional predictor for diabetes complications, based either on HbA1c or fasting plasma glucose (FPG) [7–9]. Coefficient of variation (CV) and standard deviation (SD) of HbA1c and FPG are the most commonly reported visit-to-visit measures of GV (GVs) [7, 10, 11].

Many prognostic models have been developed for diabetes complications (e.g., cardiovascular disease (CVD), diabetes retinopathy (DR) and chronic kidney disease (CKD)) using traditional statistical models [12, 13]. There has been increasing interest in the analysis of multi-dimensional healthcare data, with linear or non-linear relationships, using machine learning (ML) models in the prediction of diabetes complications [14–16]. However, published ML prognostic models have not included MGVs among their predictors. Only one study used GVs in traditional statistical models (Cox proportional hazards, CPH) and multiple logistic regression [7]; their prognostic model performance, as measured by C-statistics, ranged between 0.67 and 0.87 [12, 13]. Moreover, in resource-limited settings, particularly in developing countries where HbA1c testing is less accessible, the use of FPG-GV could be advantageous.

Hence, this study aims to assess and compare the ability of different GVs based on HbA1c or FPG, specifically CV, SD and time-varying, to predict type 2 diabetes (T2D) complications (i.e. CVD, DR and CKD); we used both traditional statistical models (CPH regression) and machine learning models (random survival forest (RSF) and left-truncated and right-censored survival (LTRC) forest).

## Methods

### Study population

**Fig. 1:**
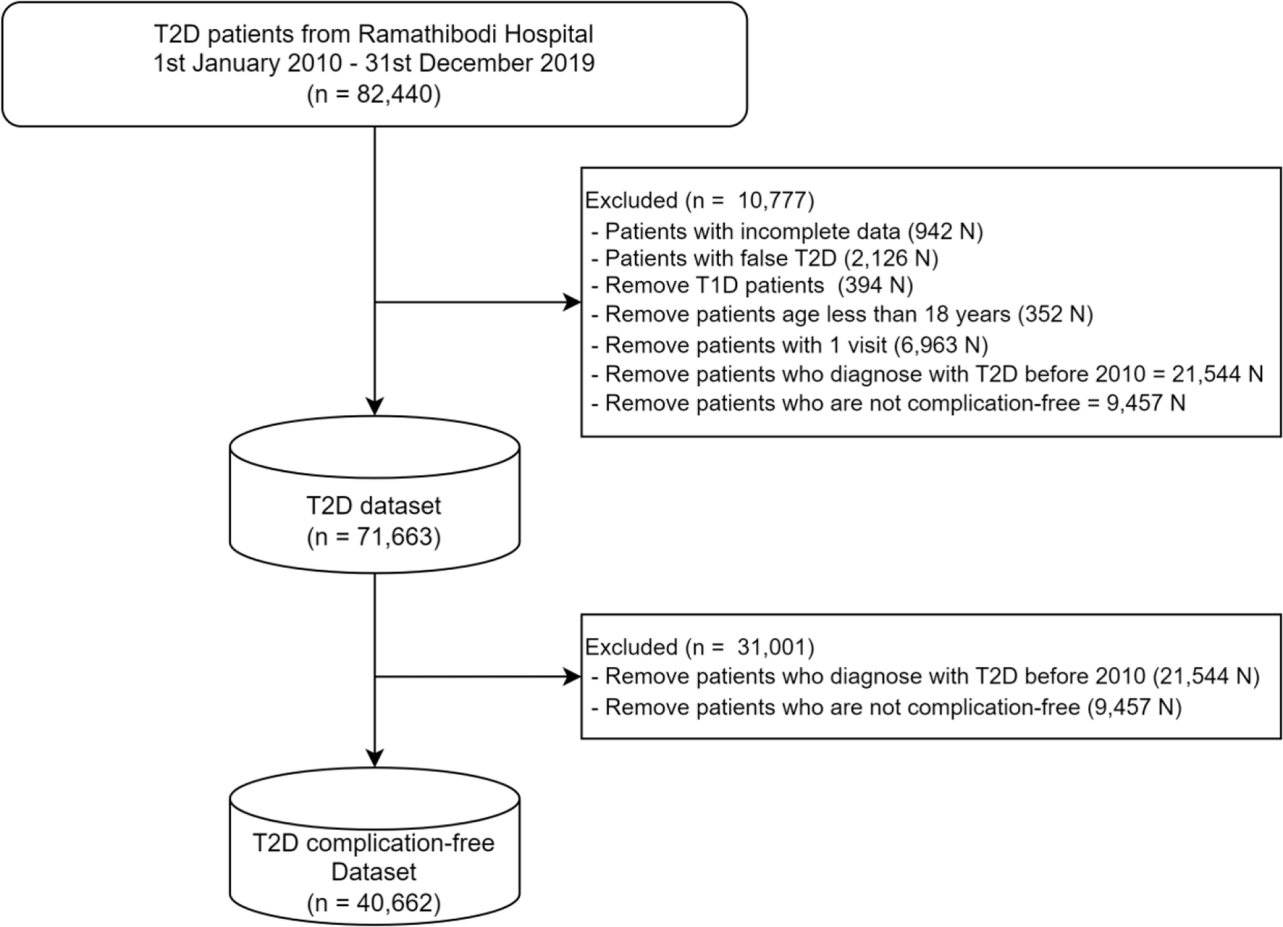
Dataflow of T2D cohort

This study utilized a retrospective cohort design, using a real-world T2D clinical cohort from the Ramathibodi Hospital, a tertiary care center in Thailand [17]. Data related to patient demographics, diagnosis, laboratory results, and medications were extracted from January 2010 to December 2019. The dataflow of cohort creation is shown in Fig. 1. Ethics approval from the Institutional Review Board of Faculty of Medicine, Ramathibodi Hospital was obtained prior to conducting the research (COA.MURA 2022/100 and COA.MURA2022/474).

Patients who were diagnosed with T2D based on International Classification of Diseases version (ICD) 10 (see Supplement Table 1), prescribed any diabetes medications (see Supplement Table 2), or who had any of the following: FPG ≥ 7.0 mmol/L in two consecutive tests, 2-hour post prandial glucose ≥ 11.1 mmol/L, or HbA1c ≥ 6.5%, were included in the study. In addition, patients had to have at least two readings of HbA1c or FPG in the first two years for the purpose of calculating GVs. Patients who were on dialysis or had the outcome of interest at the first visit were excluded from the study.

**Table 1:** Baseline patient characteristics

	*N* = 40,662
Age (years), mean (SD)	57.2 (13.9)
Male, n (%)	15,560 (38.3)
Insurance scheme, n (%)	
Civil servant scheme	16,220 (50.3)
National health insurance	3,774 (11.7)
Social security insurance	1,229 (3.8)
Others	11,043 (34.2)
Body mass index (kg/m^2^), mean (SD)	28.1 (5.7)
*Lab investigations*, mean (SD)	
Total cholesterol (mg/dL)	198.4 (63.2)
Low-density lipoprotein cholesterol (mg/dL)	129.3 (42.0)
High-density lipoprotein cholesterol (mg/dL)	47.0 (13.0)
Triglyceride (mg/dL)	171.6 (158.1)
HbA1c (%)	7.7 (2.0)
FPG (mg/dL)	153.2 (78.6)
HbA1c-CV	0.07 (0.08)
HbA1c-SD	0.67 (0.87)
FPG-CV	0.12 (0.14)
FPG-SD	25.98 (40.67)
Haemoglobin level (mg/dL)	13.21 (1.68)
Uric acid (mg/dL)	5.95 (1.80)
Systolic blood pressure (mmHg)	141.92 (20.17)
Diastolic blood pressure (mmHg)	83.14 (9.62)
*Comorbidities*, n (%)	
Hypertension	27,617 (67.9)
Hyperlipidemia	27,363 (67.3)
*Medication use*, n (%)	
Biguanides	23,615 (58.1)
Sulphonylurea	7,752 (19.1)
Insulin	3,305 (8.1)
Alpha-glucosidase inhibitors	2,540 (6.2)
Dipeptidyl peptidase-4 inhibitors	2,838 (7.0)
Glucagon-like peptide-1 agonists	138 (0.3)
Thiazolidinediones	2,290 (5.6)
Sodium-glucose cotransporter-2 inhibitors	250 (0.6)
Meglitinides	53 (0.1)
Statins	18,648 (45.9)

CV: Coefficient of variation; FPG: Fasting plasma glucose HbA1c: Haemoglobin A1c; SD: standard deviation

**Table 2:** Results of random survival forest

	Cardiovascular disease				Diabetes retinopathy				Chronic kidney disease			
	HbA1c-CV	HbA1c-SD	FPG-CV	FPG-SD	HbA1c-CV	HbA1c-SD	FPG-CV	FPG-SD	HbA1c-CV	HbA1c-SD	FPG-CV	FPG-SD
*Tuned hyperparameters*												
No. of trees (n_estimators)	256	256	32	256	256	128	128	128	64	256	32	128
Minimum samples required to split at a node (min_samples_split)	20	15	20	20	5	10	10	10	20	15	5	20
Maximum number of samples in a leaf node (max_leaf_nodes)	30	10	10	30	10	10	10	10	50	10	10	10
Maximum tree depth (max_depth)	4	8	10	4	4	4	4	4	8	8	4	12
*Model evaluation: C-index*												
Train dataset	0.764	0.761	0.772	0.770	0.835	0.833	0.844	0.847	0.845	0.782	0.775	0.784
Test dataset	0.727	0.726	0.708	0.698	0.774	0.776	0.787	0.784	0.724	0.736	0.740	0.738

Two types of dataset were created. Firstly, the “baseline dataset” used baseline covariates and “naïve method” GVs; these included HbA1c-CV, HbA1c-SD, FPG-CV and FPG-SD. GVs for the baseline dataset which were referred as “naïve method” GV measures. They were calculated using HbA1c or FPG readings in the first two years of visit. We included one GV in each of the models. The formula used for CV and SD calculations are shown below [18]:

$$\:CV=\:\frac{\frac{Standard\:deviation}{Mean}}{\sqrt{\frac{N}{N-1}}}\:;\:\:\:\text{N}=\:\text{t}\text{o}\text{t}\text{a}\text{l}\:\text{n}\text{u}\text{m}\text{b}\text{e}\text{r}\:\text{o}\text{f}\:\text{v}\text{i}\text{s}\text{i}\text{t}\text{s}$$

Secondly, a “longitudinal dataset” used time-varying FPG and HbA1c in a time to event model (described below).

### Outcomes of interest

The diabetic complications (CVD, CKD and DR) were identified using their respective ICD 9 and 10 codes, see Supplement Table 1. In addition, patients with glomerular filtration rate < 60 mL/min/1.73m^2^ for more than 3 consecutive readings in 6 months were classified as having CKD.

### Covariates

The included covariates were age, gender, insurance scheme, body mass index (BMI), total cholesterol, low-density lipoprotein (LDL), high-density lipoprotein (HDL), triglyceride, haemoglobin, systolic and diastolic blood pressure (SBP/DBP), hypertension, dyslipidemia, presence of T2D complications (CVD, DR, or CKD) prior to the outcome of interest, medication use in terms of drug classes (biguanides, sulphonylurea, insulin, alphaglucosidase inhibitors, dipeptidyl peptidase-4 inhibitors (DPP-4i), glucagon-like peptide-1 agonists (GLP1-RA), thiazolidinedione (TZD), sodium-glucose cotransporter-2 inhibitors (SGLT2i), meglitinides, statins) and the number of antihypertensive drugs.

### Models

We compared traditional statistical CPH models with ML models in both baseline and longitudinal datasets. For the baseline dataset, CPH and RSF were used, whereas for the longitudinal dataset, CPH and left-truncated right censor (LTRC) random forest were used.

The CPH model was used because it is a semi-parametric model which is commonly applied for time-to-event outcomes in medical research. It is more flexible than parametric survival models because it requires only the proportional hazards assumption. RSF and LTRC models were used due to their ability to effectively handle non-linear associations and high-order interactions among covariates. These become increasingly complex and difficult to interpret in the CPH model, particularly with more than three-way interactions.

### Data analysis

Data were reported in number and percentage for categorical variables, and mean and SD for continuous variables. We utilized survival analysis to model time-to-event data. For the baseline dataset, the patient’s first entry in the retrospective cohort was taken. All the visits were included in the longitudinal dataset for time-varying GVs. For modeling and evaluation, the data were divided into training and test sets in a 70:30 ratio.

A CPH model was applied by regressing each GV (i.e., GV-SD and GV-CV) on complication for baseline data, whereas time-varying GV was used for longitudinal data. A total of 26 covariates were also considered for feature selection: First, a simple CPH model was constructed by fitting each of covariates in the model. Second, a multivariate CPH model was performed simultaneously including covariates whose p-values < 0.2 in the first step. Only clinical and/or statistically significant covariates (p-value < 0.05) by a likelihood ratio test were retained in the final model [19]. Adjusted hazard ratios (HR) along with 95% confidence interval (CI) were estimated and reported. Schoenfeld residuals were used to check for the proportional hazard assumption.

For ML models, RSF was used with the baseline dataset to find the association between either GV-CV or GV-SD and diabetic complications. Covariates with a univariate CPH p-value < 0.2 were simultaneously included in the RSF model alongside GV-CV and GV-SD. Internal feature selection was performed based on feature importance and model optimization. Hyperparameter tuning for RSF was performed using the “RandomizedSearchCV” function in sklearn package [20].

Longitudinal data were analyzed using a random-effect CPH model and LTRC forest incorporating time-varying GVs and time-varying confounders. Feature selection mirrored the baseline analysis. Hyperparameter tuning for LTRC forest was performed manually as there was no readily available function, see more details in Supplement Table 3. Model performance was evaluated with C-statistic, which measured model discriminative performance and classified as poor, fair, good, and excellence if it was < 0.6, 0.6-<0.7, 0.7- <0.8, ≥ 0.8, respectively [21].

**Table 3:** The model performance using the C-index for each complication and glycemic variability in the test set

Complication	GV	Models		
		CPH	RSF	LTRC-forest
CVD	HbA1c-CV	0.704	0.727	-
	HbA1c-SD	0.703	0.726	-
	HbA1c-TV	0.730	-	0.468
	FPG-CV	0.709	0.708	-
	FPG-SD	0.706	0.698	-
	FPG-TV	0.730	-	0.484
DR	HbA1c-CV	0.755	0.774	-
	HbA1c-SD	0.753	0.776	-
	HbA1c-TV	0.758	-	0.612
	FPG-CV	0.754	0.787	-
	FPG-SD	0.748	0.784	-
	FPG-TV	0.748	-	0.678
CKD	HbA1c-CV	0.735	0.724	-
	HbA1c-SD	0.734	0.736	-
	HbA1c-TV	0.750	-	0.579
	FPG-CV	0.739	0.740	-
	FPG-SD	0.736	0.738	-
	FPG-TV	0.750	-	0.555

The integrated Brier score was also used to assess the calibration of LTRC forest, where 0 reflects the model is well calibrated. All analyses were performed based on complete case data. CPH was performed using R^®^ software (survival package v3.6). RSF was performed using Python^®^ 3.8 with sksurv package version 0.17.1 and LTRC forest v0.7.0. P-value of less than 0.05 was considered as statistically significant.

## Results

A total of 40,662 patients with T2D fulfilled our eligibility criteria between 2010 and 2019; most were female (61.7%), with a mean age of 57.2 years, were covered by the government insurance scheme (50.3%), and were obese (mean body mass index 28.1 kg/m2), see Table 1. FPG and HbA1c were measured approximately every 3–6 month interval with overall mean within two-year interval of 153.2 (78.6) and 7.7 (2.0), respectively. A total of 3,921 (9.6%), 2,305 (5.7%), and 4,594 (11.3%) patients developed CVD, DR, and CKD respectively.

### CVD

**Fig. 2:**
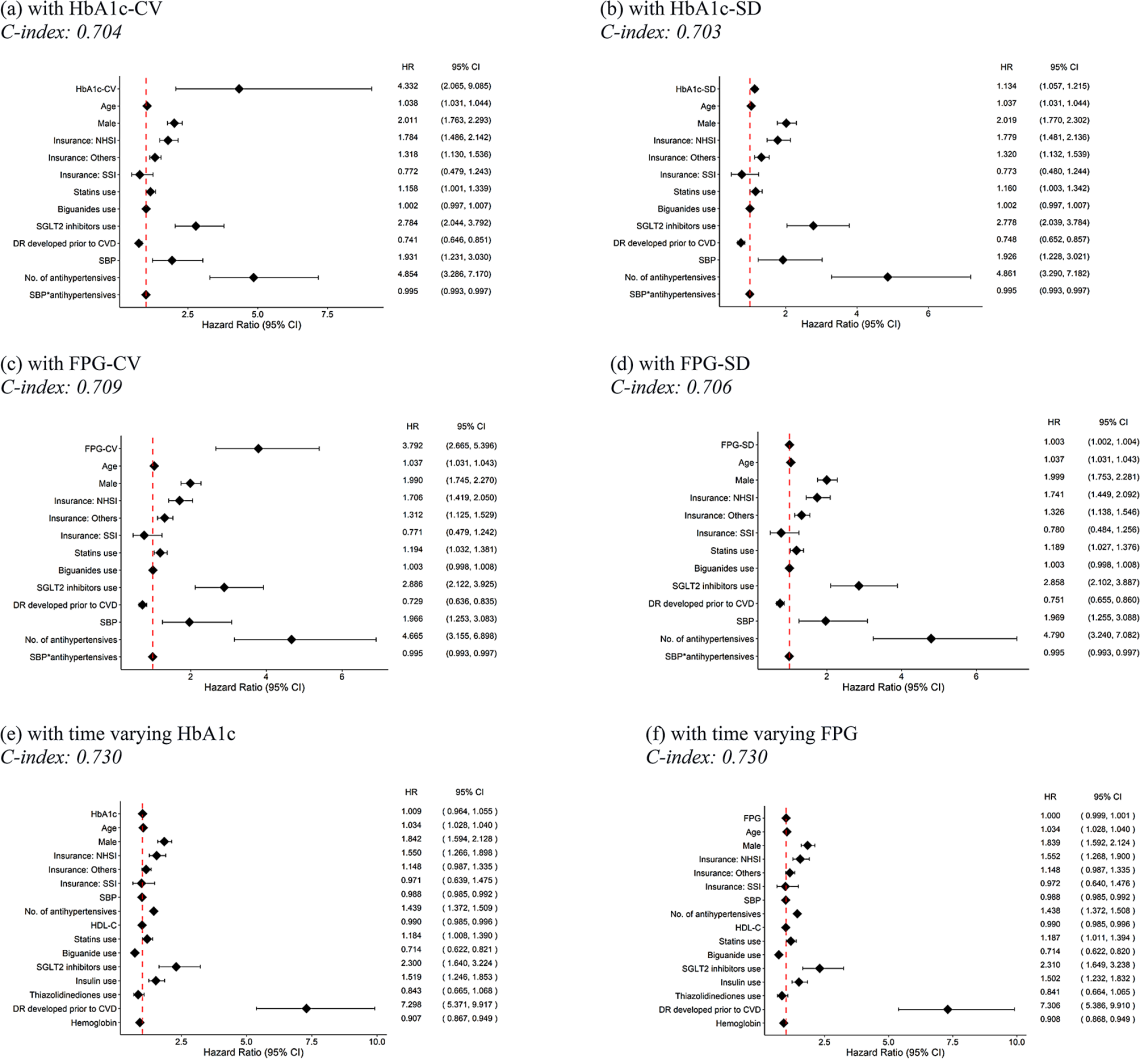
Hazard ratios for cardiovascular disease (CVD) outcomes. BMI: Body mass index; CI: confidence interval; CKD: Chronic kidney disease; CV: Coefficient of variation; CVD: Cardiovascular disease; DR: Diabetic retinopathy; DPP-4: Dipeptidyl peptidase 4; FPG: Fasting plasma glucose; HbA1c: Haemoglobin A1c; HDL-C: high-density lipoprotein cholesterol; HR: hazard ratio; NHSI: National health insurance; SBP: Systolic blood pressure; SD: standard deviation; SGLT2: sodium-glucose cotransporter-2; SSI: Social security insurance

Of 40,662 in the complete-case dataset, 3,921 patients developed CVD. For baseline data, a simple CPH model was applied to all four “naïve method” GVs (i.e. HbA1c-CV, HbA1c-SD, FPG-CV and FPG-SD), and 23 out of 26 covariates were shown to be significant, see Supplementary Table 4. Each of GVs plus 23 covariates were simultaneously included in multivariate CPH models, see Fig. 2a and d. All the “naïve method” GVs were significantly associated with CVD but HbA1c-CV yielded highest HR following by FPG-CV with the HRs (95% CI) of 4.332 (2.065, 9.085) and 3.792 (2.665, 5.396), respectively; whereas FPG-SD was poorestly performed with HR of 1.003 (1.002–1.004), as judges by the HRs. In addition, male, age, use of statins and SGLT2i, DR at baseline, SBP and number of antihypertensives were also associated with increased risk of developing CVD. The use of biguanides, on the other hand, was associated with a decreased risk of CVD. C-indices for HbA1c-CV, HbA1c-SD, FPG-CV and FPG-SD in the training set were 0.722, 0.721, 0.728, and 0.725, respectively, while those in the test set were 0.704, 0.703, 0.709, and 0.706.

The RSF models were constructed based on selected hyperparameters and their performance is shown in Table 2. The C-indices in the training set for HbA1c-CV, HbA1c-SD, FPG-CV, and FPG-SD models were 0.764, 0.761, 0.772 and 0.770; these corresponding values in the test set were 0.727, 0.726, 0.708, and 0.698. However, permutation feature importance suggested that HbA1c-CV, HbA1c-SD, FPG-CV and FPG-SD were ranked at 17, 17, 12 and 11 out of 23, respectively (see Supplement Table 5). FPG GVs had higher concordance and also ranked higher in permutation importance as important variables.

For the longitudinal dataset, CPH models were constructed by fitting time-varying HbA1c and FPG in the model indicating both features were not significant, with HRs (95% CI) of 1.009 (0.964, 1.055) and 1.000 (0.999, 1.001), see Fig. 2e f. In addition, male, age, use of statins, insulin and SGLT2i, baseline DR, and number of antihypertensives were also associated with CVD risk. Conversely, high SBP, HDL, haemoglobin, and the use of biguanides were associated with decreased CVD risk. C-indices for these two corresponding CPH models in training and test sets were 0.726 and 0.730; and 0.726 and 0.730, respectively.

LTRC forest was manually tuned with a selected number of trees and tree depth of 16 and 6, respectively. LTRC forest results showed that increasing the number of trees could improve the integrated Brier score and concordance, but at the same time, the training time increased substantially, see Supplement Table 6. However, increasing node depth increased the difference in integrated Brier score and concordance between training and test sets, which is a sign of model overfitting. Discrimination performance was poor in both HbA1c and FPG models with the C-index for the training and test sets of 0.534 and 0.468; and 0.564 and 0.484, respectively.

Discriminative performance for all models is summarized in Table 3. Comparing different GVs in the CPH models, HbA1c-TV (time-varying) yielded higher discrimination than HbA1c-SD and Hb1A1c-CV. In addition, FPG-TV had similar result HbA1C-TV and were slightly better than RSF, but much better than LTRC forest model.

### DR

**Fig. 3:**
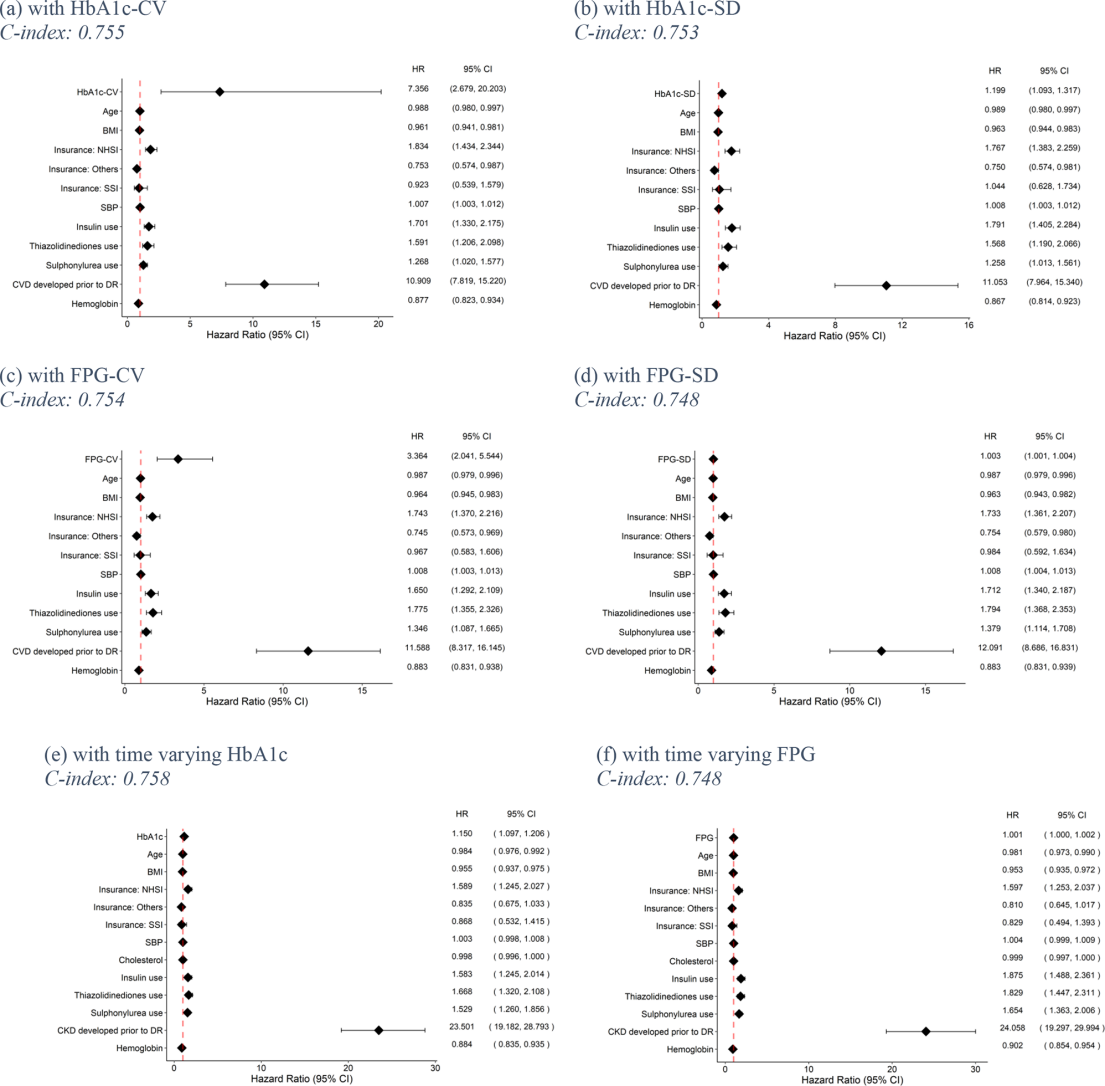
Hazard ratios for diabetes retinopathy (DR) outcomes. BMI: Body mass index; CI: confidence interval; CKD: Chronic kidney disease; CV: Coefficient of variation; CVD: Cardiovascular disease; DR: Diabetes retinopathy; DPP-4: Dipeptidyl peptidase 4; FPG: Fasting plasma glucose; HbA1c: Haemoglobin A1c; HDL-C: high-density lipoprotein cholesterol; HR: hazard ratio; NHSI: National health insurance; SBP: Systolic blood pressure; SD: standard deviation; SGLT2: sodium-glucose cotransporter-2; SSI: Social security insurance

Of 40,662 in the complete-case dataset, 2,305 patients developed DRs. A simple CPH model was applied to all four “naïve method” GVs (i.e. HbA1c-CV, HbA1c-SD, FPG-CV and FPG-SD), and 20 of 26 covariates were shown to be significant, see Supplementary Table 4. Figure 3a and d show the summary of all HRs indicating all the “naïve method” GVs were significantly associated with DR. Increase in glucose variability was associated with an increased risk of developing DR, with hazard ratios (95% CI) of 7.356 (2.679, 20.203), 1.199 (1.093, 1.317), 3.364 (2.041, 5.544), and 1.003 (1.001–1.004) for HbA1c-CV, HbA1c-SD, FPG-CV, FPG-SD, respectively. In addition, SBP, use of insulin, TZD, sulphonylurea, and CVD at baseline were associated with an increased risk of developing DR. Conversely, increase in age, BMI and haemoglobin were associated with a decreased risk of DR. C-indices for HbA1c-CV, HbA1c-SD, FPG-CV and FPG-SD in training set were 0.739, 0.739, 0.737, and 0.740, respectively, while those in the test set were 0.755, 0.753, 0.754, and 0.748.

For RSF models, C-indices of respective models in the training set for HbA1c-CV, HbA1c-SD, FPG-CV, and FPG-SD were 0.835, 0.833, 0.844 and 0.847 whereas C-indices for these corresponding models in the test set were 0.774, 0.776, 0.787 and 0.784, see Table 3. Permutation feature importance showed that all GVs were ranked as the 1st out of 21 features (see Supplement Table 5).

For the longitudinal dataset, both time-varying HbA1c and FPG were significantly associated with DR in their respective CPH models, with HRs (95% CI) of 1.150 (1.097, 1.206) and 1.001 (1.000, 1.002), see Fig. 3e f. In addition, the use of sulphonylurea, insulin, TZD, and prior CVD were associated with increased risk of developing DR whereas age, BMI and haemoglobin were associated with a decreased risk of DR. C-indices for the model with time-varying HbA1c and FPG in training and test sets were 0.794 and 0.758: and 0.781 and 0.748, respectively.

LTRC forest was manually tuned with a selected number of trees and tree depth of 16 and 10 for the HbA1c model and 32 and 10 for the FPG model. Increasing the number of trees improved the integrated Brier score and concordance, but at the same time, the time for building the model increased to as high as 5.7 h, see Supplement Table 6. An increase in the node depth, however, increased the difference in integrated Brier score and concordance between training and test sets, again indicating overfitting. C-indices in the training and test sets were 0.823 and 0.612 for HbA1c models, and 0.812 and 0.678 for FPG models.

Among the CPH models, most HbA1c yielded better discrimination than FPG, but they performed more poorly relative to HbA1c -CV and HbA1c -SD in the RSF models, see Table 3.

### CKD

**Fig. 4:**
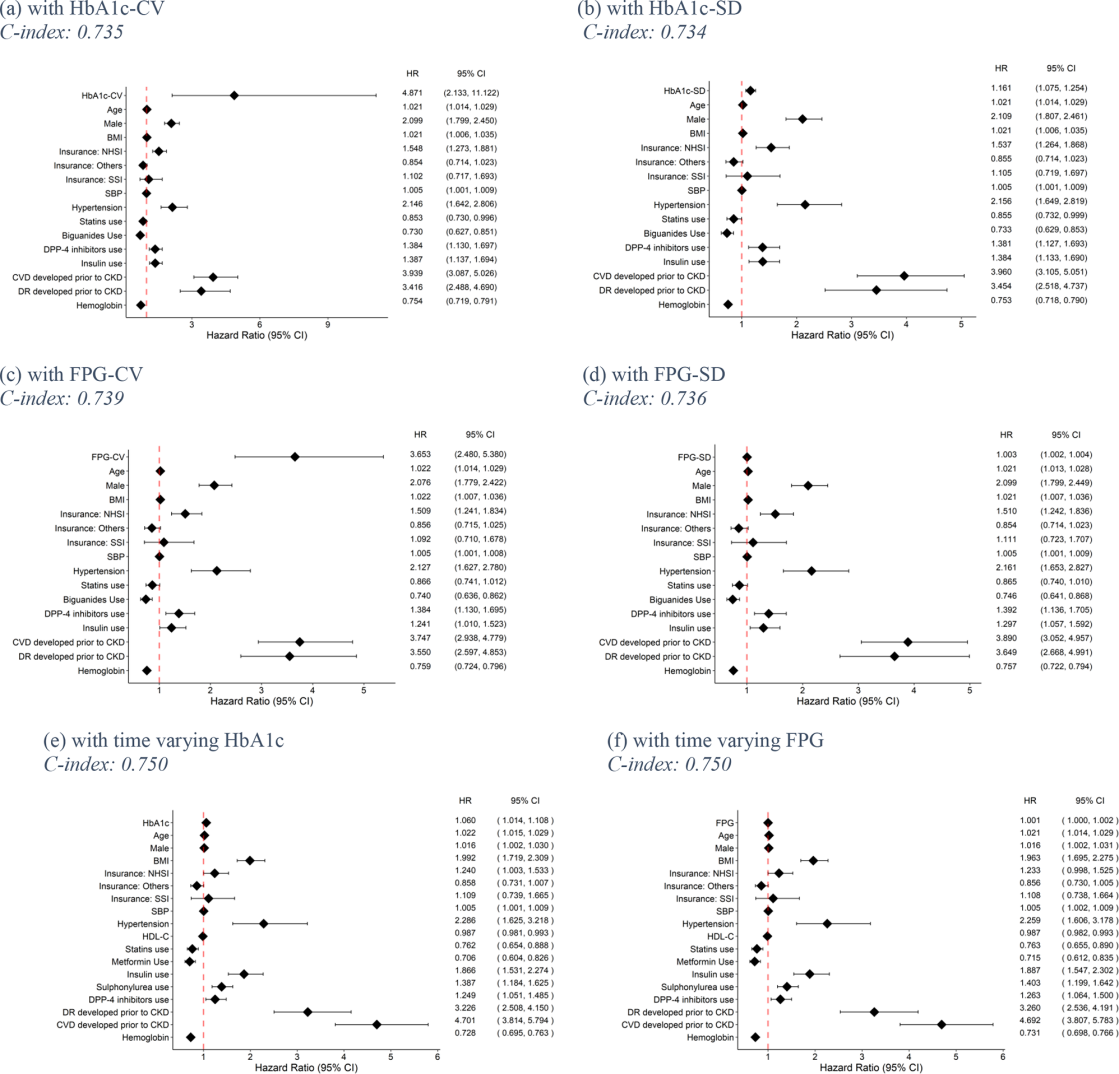
Hazard ratios for chronic kidney disease (CKD) outcomes. BMI: Body mass index; CI: confidence interval; CKD: Chronic kidney disease; CV: Coefficient of variation; CVD: Cardiovascular disease; DR: Diabetes retinopathy; DPP-4: Dipeptidyl peptidase 4; FPG: Fasting plasma glucose; HbA1c: Haemoglobin A1c; HDL-C: high-density lipoprotein cholesterol; HR: hazard ratio; NHSI: National health insurance; SBP: Systolic blood pressure; SD: standard deviation; SGLT2: sodium-glucose cotransporter-2; SSI: Social security insurance

Of 40,662 in the complete-case dataset, 4,594 patients developed CKD. Simple CPH of four “naïve method” GVs (i.e. HbA1c-CV, HbA1c-SD, FPG-CV and FPG-SD) and 23 of 26 covariates were significant, Supplementary Table 4. All the “naïve method” GVs were significant in the multivariable CPH models (see Fig. 4a and d) with hazard ratios (95% CI) of 4.871 (2.133, 11.122), 1.161 (1.075, 1.254), 3.653 (2.480, 5.380), 1.003 (1.002, 1.004) for HbA1c-CV, HbA1c-SD, FPG-CV, FPG-SD, respectively. Age, male, BMI SBP, hypertension, use of insulin and DPP-4i, and prior CVD and DR, were associated with increased CKD risk. Biguanides use and haemoglobin were associated with a decreased risk of CKD. C-indices for HbA1c-CV, HbA1c-SD, FPG-CV and FPG-SD in training set were 0.746, 0.746, 0.752, and 0.748, respectively, while in test set were 0.735, 0.734, 0.739, and 0.736.

RSF models yielded C-indices in the training dataset for HbA1c-CV, HbA1c-SD, FPG-CV, and FPG-SD of 0.845, 0.782, 0.775 and 0.784; these corresponding values in the test set were 0.724, 0.736, 0.740, and 0.738, see Table 3. Permutation feature importance showed that HbA1c-CV, HbA1c-SD, FPG-CV and FPG-SD were ranked at 8, 7, 6 and 3 out of 24 features, respectively (see Supplement Table 5).

CPH models of both time-varying HbA1c and FPG were not significantly associated with CKD, with HRs (95% CI) of 1.060 (1.014, 1.108) and 1.001 (1.000, 1.002), see Fig. 4e f. Moreover, age, male, BMI, SBP, hypertension, use of sulphonylurea, insulin and DPP4-i, and prior CVD or DR were associated with increased CKD risk whereas haemoglobin and statins use were associated with decreased CKD risk. C-indices in training and test sets were 0.776 and 0.750 for the model using time-varying HbA1c, while 0.775 and 0.750 for the time-varying FPG model.

LTRC forest was manually tuned and a selected number of trees and tree depth of 32 and 10 for the HbA1c model, and 16 and 10 for the FPG model, see Supplement Table 6. Increasing the number of trees improved the integrated Brier score and concordance, but the training time increased. An increase in the node depth indicated model overfitting again, with the C-indices for HbA1c model in the training and test sets of 0.717 and 0.579, and for the FPG model of 0.705 and 0.555.

Comparing discriminative performance among different GVs indicated that FPG-TV and HbA1c-TV were better than FPG-CV in the CPH models. However, all the CPH models performed better than the RSF and LTRC-forest models, see Table 3.

## Discussion

We conducted the study to assess which GVs could best predict complications of T2D. Based on baseline data, all four GVs (i.e., HbA1c-CV, HbA1c-SD, FPG-CV and FPG-SD) were associated with all three diabetes complications of CVD, DR and CKD with well discriminate performance, whereas time-varying HbA1c and FPG were only associated with DR and CKD with poor and fair discriminate performance [21, 22], respectively. Among the three complications, the DR and CKD models had marginally better performance than CVD models. Among the GVs, CPH models using FPG-CV achieved the highest c-indices while feature importance in RSF ML indicated that FPG-SD was ranked highest in predicting T2D-complications, followed closely by FPG-CV. The RSF ML model performed slightly better than the CPH model for DR. The models based on longitudinal data showed again marginally better performance in prediction of DR and CKD but not for CVD than models based on baseline data. Overall, the best models for the prediction of CVD, DR, and CKD were FPG-CV with CPH, FPG-CV with RSF, and FPG-TV with CPH, respectively. Permutation feature importance indicated that GV might be most useful for predicting DR (first rank) and CKD (third rank) compared to CVD (11th rank).

GVs based on HbA1c and FPG were very close in prognostic performance. Unmet needs for diabetes cascade of care (i.e. testing, diagnosis, treatment and control) in 28 low- and middle-income countries was shown to be high, which indicates a need for improvement [23]. In terms of diabetes monitoring, a study in Thailand showed that FPG was more widely used than HbA1c (89.1s% versus 76.8%) [24]. We postulate that HbA1c might not be widely used or monitored in resource-limited settings due to the cost constraints. Hence, the use of actual FPG, FPG-CV, or FPG-SD could be a reasonable substitute for HbA1c in diabetes monitoring and prognostication, and may even show advantages among patients with diabetes and CKD as anemia is common among CKD patients, which will affect the reliability of HbA1c [25].

Published prognostic models for DR and CKD showed that C-indices for DR are generally higher than CKD in the development phase, internal validation phase and external validation phase (DR: 0.82, 0.83, 0.81 vs. CKD: 0.78, 0.79, 0.75) [13]. For prognostic models for CVD, studies reported C-indices ranging from 0.64 to 0.80, with a pooled C-index of 0.67 [12]. DR and CKD prognostic models have better performance as compared to CVD models. One possible explanation is that CVD has a more complex mechanism or pathophysiology. Thus, further studies need to explore other risk factors that could be included to improve the predictive ability of CKD and CVD models. In our study, DR models developed using RSF performed the best, but these still need external validation.

The HRs estimated based on baseline data for HbA1c-CV, HbA1c-SD, FPG-CV, and FPG-SD are generally higher than those obtained using time-varying HbA1c or FPG in the longitudinal dataset. In other words, the effect sizes are “diluted” when estimated based on longitudinal data. This is not surprising because the longitudinal data takes into account all changes of these GVs over time during follow up. When we performed proportionality assumption checks on the GVs, we observed that the assumption was sometimes violated in the baseline dataset (all GVs in CKD models; HbA1c-CV and HbA1c-SD from DR models) and in the longitudinal dataset (only FPG in CKD model). Thus, the longitudinal dataset is more reliable as most of the models in the baseline dataset did not fulfill the proportional hazard assumption. This may explain the higher C-indices in the longitudinal dataset for DR and CKD models compared to baseline dataset models. On the other hand, if there is only baseline data, RSF models are more reliable when there is a violation of the proportional hazard assumption.

One of the strengths of this study is that we utilized several MGVs (naïve method and time-varying) to explore the predictive ability of each GV measure on three diabetes complications. Furthermore, we utilized two different datasets – baseline and longitudinal, where the baseline dataset requires less computational power but the longitudinal dataset included time-varying covariates which can handle non-proportional hazards. We also included two different ML models to compare the performance with CPH regression. As a result, we identified that FPG was a better parameter than HbA1c, and FPG-CV in the CPH or RSF models performed best for clinical prognostication. Nonetheless, this study has its limitations. The ML models, particularly the LTRC forest, showed evidence of overfitting, with substantial discrepancies between training and testing C-indices. To mitigate this, further model tuning and validation are required using expanded datasets (internal data with increased observations and external datasets from diverse settings). Furthermore, the single-center design, based on a tertiary care hospital in Thailand, limits the generalizability of our findings to other populations and healthcare systems, both within and outside of Asia. External validation is therefore necessary.

## Conclusions

Different modalities were used to create GVs – using both naïve and time-varying methods. GVs using both HbA1c and FPG performed similarly; hence, FPG GV can be used as a monitoring and prognostic parameter when HbA1c is unavailable or less accessible. The best models for predicting CVD, DR and CKD used FPG-CV in either CPH or RSF models.

## Electronic supplementary material

Below is the link to the electronic supplementary material.

Supplementary Material 1

## Data Availability

Access to the dataset from the current study is available from the corresponding author upon reasonable request and approval.

## Abbreviations

CVD: Cardiovascular disease
CPH: Cox proportional hazards
CV: Coefficient of variation
CKD: Chronic kidney disease
DPP-4i: Dipeptidyl peptidase-4 inhibitors
DR: Diabetic retinopathy
FPG: Fasting plasma glucose
GLP1-RA: Glucagon-like peptide-1 agonists
GV: Glucose variability
HbA1c: Haemoglobin A1c
HR: Hazard ratios
LTRC: Left-truncated, right-censored
RSF: Random survival forest
SD: Standard deviation
SGLT2i: Sodium-glucose cotransporter-2 inhibitors
T2D: Type 2 diabetes
TZD: Thiazolidinedione
