# Supplementary material for: Predictive ability of visit-to-visit glucose variability on diabetes complications
Source: BMC Med Inform Decis Mak. 2025 Mar 17;25:134. doi: 10.1186/s12911-025-02964-2 (PMC11917057; doi:10.1186/s12911-025-02964-2)
Supplement: Supplementary file 1 — Supplementary Material 1 [file 12911_2025_2964_MOESM1_ESM.docx]

**Supplement tables**

Supplement table 1: International Classification of Diseases codes for type 2 diabetes and its complications

Supplement table 2: Medication for type 2 diabetes

Supplement table 3: Hyperparameter tuning

Supplement table 4: Simple Cox regression

Supplement table 5: Random Survival Forest Permutation Importance Top 20 Ranking

Supplement table 6: Left-truncated, right-censored (LTRC) Forest results

Supplement table 1: International Classification of Diseases codes for type 2 diabetes and its complications

| **Features** | **Description** |
| --- | --- |
| Type 2 diabetes | **ICD-10**: E11* |
| Diabetes retinopathy | **ICD-10:** E11.32, E11.33, E11.34, E11.35, H28.0, H36.0 |
| Chronic kidney disease | **ICD-10:** N18.1, N18.2, N18.3, N18.4, N18.5, N18.9, E11.2, N08.3  **ICD-9:** 3895, 3927, 3942, 3943,3995, 5498  **Lab:** eGFR < 60 for 3 consecutive times in 6 months |
| Cardiovascular disease | **ICD-10:** I20-I25, I50.0, I50.1, I50.9, I51.6, I51.7, I60-I69, G46  **ICD-9:** 0040, 0041, 0042, 0043, 0044, 0045, 0046, 0047, 0048, 3600-3605, 3610-3617, 3619, 3732, 3810-3816, 3818 |

ICD-9: International Classification of Diseases version 9; ICD-10: International Classification of Diseases version 10

Supplement table 2: Medication for type 2 diabetes

| **Class** | **Medications that are included** |
| --- | --- |
| Sulphonlyurea | Chlorpropamide, Glibenclamide, Gliclazide,  Glimepiride, Glipizide, Glyburide, Tolazamide,  Tolbutamide |
| Alpha-glucosidase inhibitors | Acarbose, Miglitol, Voglibose |
| Biguanides | Glucophage/ Metformin |
| Dipeptidyl-peptidase-4 inhibitors | Alogliptin, Gemigliptin, Linagliptin, Saxagliptin, Sitagliptin, Vildagliptin |
| Thiazolidinedione | Pioglitazone, Rosiglitazone |
| Sodium-glucose cotransporter-2 inhibitors | Canagliflozin, Dapagliflozin, Empagliflozin, Ertugliflozin, Luseogliflozin |
| Glucagon-like peptide-1 agonist | Dulaglutide, Exenatide, Liraglutide, Lixisenatide, Semaglutide |
| Insulin | Aspart, Degludec, Detemir, Glargine, Glulisine, Insulin inhalation, Insulin isophane, Lispro, Regular |
| Meglitinides | Nateglinide, Repaglinide |

# Supplement table 3: Hyperparameter tuning

| **Random Survival Forest** | | |
| --- | --- | --- |
| 1 | Number of trees in the forest (“n_estimators”) | 32, 64, 128, 256 |
| 2 | Minimum number of samples required to split an internal node (“min_samples_split”) | 5, 10, 15, 20 |
| 3 | Maximum number of samples in a node (“max_leaf_nodes”) | 10, 20, 30, 40, 50 |
| 4 | Maximum depth of a tree (“max_depth”) | 4, 8, 10, 12 |
| **Number of parameters setting that were sampled was fixed at 100 and 5-fold cross validation was performed*  **The best parameters were chosen based on the narrower gap between mean train score and mean test score, which is an attempt to avoid overfitting* | | |
|  | | |
| **LTRC Forest** | | |
| 1 | Number of variables to try at each potential split (“mtry”) | Determined by tuning the model and identify the best “mtry”, i.e., the smallest out-of-bag error |
| 2 | Number of trees (“ntree”) | 16, 32, 64 |
| 3 | Tree depth (“nodedepth”) | 6, 8, 9, 10 |

Supplement table 4: Simple Cox regression

| **Variable** | **n** | **Hazard ratio** | **SE** | **95% CI** | **P-value** |  |
| --- | --- | --- | --- | --- | --- | --- |
| **Cardiovascular disease (CVD)** | | | | | | |
| HbA1c-CV | 24,168 | 0.552 | 0.25 | 0.339, 0.900 | 0.017 |  |
| HbA1c-SD | 24,168 | 0.930 | 0.024 | 0.888, 0.975 | 0.002 |  |
| FPG-CV | 30,279 | 3.442 | 0.105 | 2.804, 4.226 | <0.001 |  |
| FPG-SD | 30,279 | 1.003 | 0.001 | 1.003, 1.004 | <0.001 |  |
| Age | 40,662 | 1.052 | 0.002 | 1.049, 1.055 | <0.001 |  |
| Male | 40,662 | 1.829 | 0.033 | 1.718, 1.948 | <0.001 |  |
| Insurance scheme | 32,266 |  |  |  |  |  |
| Civil servant (reference) |  | 1 |  |  |  |  |
| National health insurance |  | 1.461 | 0.05 | 1.327, 1.609 | <0.001 |  |
| Social security insurance |  | 0.637 | 0.105 | 0.519, 0.782 | <0.001 |  |
| Others |  | 0.948 | 0.037 | 0.883, 1.019 | 0.144 |  |
| BMI | 22,199 | 0.976 | 0.005 | 0.967, 0.986 | <0.001 |  |
| Total Cholesterol | 31,168 | 0.997 | 0.001 | 0.996, 0.998 | <0.001 |  |
| LDL | 27,126 | 0.990 | 0.001 | 0.989, 0.991 | <0.001 |  |
| High-density lipoprotein cholesterol | 25,257 | 0.981 | 0.002 | 0.977, 0.984 | <0.001 |  |
| Triglyceride | 28,078 | 0.9996 | 0.001 | 0.9993, 0.9999 | 0.012 |  |
| Hemoglobin | 23,779 | 0.913 | 0.012 | 0.893, 0.935 | <0.001 |  |
| SBP | 23,661 | 1.008 | 0.002 | 1.006, 1.011 | <0.001 |  |
| DBP | 23,662 | 0.979 | 0.003 | 0.973, 0.984 | <0.001 |  |
| Hypertension | 40,662 | 3.159 | 0.048 | 2.875, 3.470 | <0.001 |  |
| Hyperlipidemia | 40,662 | 1.595 | 0.041 | 1.473, 1.728 | <0.001 |  |
| DR developed prior to CVD | 40,662 | 9.834 | 0.082 | 8.378, 11.540 | <0.001 |  |
| CKD developed prior to CVD | 40,662 | 11.600 | 0.047 | 10.600, 12.700 | <0.001 |  |
| Biguanides use | 40,662 | 0.708 | 0.033 | 0.665, 0.754 | <0.001 |  |
| Sulphonylurea use | 40,662 | 1.172 | 0.039 | 1.086, 1.265 | <0.001 |  |
| Insulin use | 40,662 | 1.973 | 0.05 | 1.790, 2.174 | <0.001 |  |
| Alpha-glucosidase inhibitors use | 40,662 | 0.228 | 0.146 | 0.171, 0.303 | <0.001 |  |
| Dipeptidyl peptidase-4 inhibitors use | 40,662 | 1.532 | 0.055 | 1.376, 1.706 | <0.001 |  |
| Glucagon-like peptide-1 agonists use | 40,662 | 0.498 | 0.448 | 0.207, 1.198 | 0.120 |  |
| Thiazolidinediones use | 40,662 | 0.936 | 0.072 | 0.813, 1.077 | 0.354 |  |
| Sodium-glucose cotransporter-2 use | 40,662 | 1.731 | 0.19 | 1.193, 2.511 | 0.004 |  |
| Meglitinides use | 40,662 | 1.101 | 0.448 | 0.458, 2.646 | 0.830 |  |
| Statins use | 40,662 | 1.648 | 0.034 | 1.545, 1.759 | <0.001 |  |
| Number of antihypertensives | 40,662 | 1.460 | 0.012 | 1.429, 1.492 | <0.001 |  |
| **Diabetes retinopathy (DR)** | | | | | | |
| HbA1c-CV | 24,168 | 54.590 | 0.227 | 35.010, 85.130 | <0.001 |  |
| HbA1c-SD | 24,168 | 1.424 | 0.02 | 1.369, 1.480 | <0.001 |  |
| FPG-CV | 30,279 | 12.560 | 0.111 | 10.120, 15.590 | <0.001 |  |
| FPG-SD | 30,279 | 1.006 | 0.001 | 1.006, 1.007 | <0.001 |  |
| Age | 40,662 | 1.006 | 0.002 | 1.003, 1.009 | <0.001 |  |
| Male | 40,662 | 1.226 | 0.043 | 1.129, 1.332 | <0.001 |  |
| Insurance scheme | 32,266 |  |  |  |  |  |
| Civil servant (reference) |  | 1 |  |  |  |  |
| National health insurance |  | 2.020 | 0.06 | 1.796, 2.272 | <0.001 |  |
| Social security insurance |  | 1.028 | 0.114 | 0.822, 1.285 | 0.809 |  |
| Others |  | 0.813 | 0.052 | 0.735, 0.900 | <0.001 |  |
| BMI | 22,199 | 0.954 | 0.007 | 0.942, 0.967 | <0.001 |  |
| Total Cholesterol | 31,168 | 1.001 | 0.001 | 1.000, 1.001 | <0.001 |  |
| LDL | 27,126 | 1.001 | 0.001 | 0.9996, 1.002 | 0.180 |  |
| High-density lipoprotein cholesterol | 25,257 | 0.991 | 0.003 | 0.986, 0.995 | <0.001 |  |
| Triglyceride | 28,078 | 1.000 | 0.001 | 1.000, 1.001 | 0.021 |  |
| Hemoglobin | 23,779 | 0.879 | 0.017 | 0.851, 0.907 | <0.001 |  |
| SBP | 23,661 | 1.013 | 0.002 | 1.010, 1.016 | <0.001 |  |
| DBP | 23,662 | 0.997 | 0.004 | 0.990, 1.004 | 0.389 |  |
| Hypertension | 40,662 | 1.154 | 0.047 | 1.053, 1.265 | 0.002 |  |
| Hyperlipidemia | 40,662 | 0.785 | 0.046 | 0.718, 0.859 | <0.001 |  |
| CVD | 40,662 | 19.360 | 0.073 | 16.790, 22.320 | <0.001 |  |
| CKD | 40,662 | 20.730 | 0.057 | 18.540, 23.180 | <0.001 |  |
| Biguanides use | 40,662 | 1.219 | 0.043 | 1.122, 1.325 | <0.001 |  |
| Sulphonylurea use | 40,662 | 2.616 | 0.044 | 2.403, 2.847 | <0.001 |  |
| Insulin use | 40,662 | 3.224 | 0.057 | 2.888, 3.599 | <0.001 |  |
| Alpha-glucosidase inhibitors use | 40,662 | 0.250 | 0.191 | 0.172, 0.363 | <0.001 |  |
| Dipeptidyl peptidase-4 inhibitors use | 40,662 | 1.803 | 0.068 | 1.580, 2.057 | <0.001 |  |
| Glucagon-like peptide-1 agonists use | 40,662 | 0.742 | 0.501 | 0.278, 1.980 | 0.552 |  |
| Thiazolidinediones use | 40,662 | 2.422 | 0.065 | 2.134, 2.747 | <0.001 |  |
| Sodium-glucose cotransporter-2 use | 40,662 | 1.851 | 0.252 | 1.131, 3.030 | 0.014 |  |
| Meglitinides use | 40,662 | 1.609 | 0.501 | 0.603, 4.290 | 0.342 |  |
| Statins use | 40,662 | 0.948 | 0.042 | 0.873, 1.029 | 0.200 |  |
| **Chronic kidney disease (CKD)** | | | | | | |
| HbA1c-CV | 24,168 | 6.021 | 0.192 | 4.135, 8.765 | <0.001 |  |
| HbA1c-SD | 24,168 | 1.170 | 0.018 | 1.131, 1.212 | <0.001 |  |
| FPG-CV | 30,279 | 7.031 | 0.087 | 5.939, 8.324 | <0.001 |  |
| FPG-SD | 30,279 | 1.005 | 0.001 | 1.005, 1.006 | <0.001 |  |
| Age | 40,662 | 1.045 | 0.002 | 1.043, 1.048 | <0.001 |  |
| Male | 40,662 | 1.711 | 0.030 | 1.614, 1.813 | <0.001 |  |
| Insurance scheme | 32,266 |  |  |  |  |  |
| Civil servant (reference) |  | 1 |  |  |  |  |
| National health insurance |  | 1.458 | 0.047 | 1.331, 1.597 | <0.001 |  |
| Social security insurance |  | 0.686 | 0.094 | 0.571, 0.824 | <0.001 |  |
| Others |  | 0.895 | 0.035 | 0.837, 0.958 | 0.001 |  |
| BMI | 22,199 | 0.977 | 0.005 | 0.969, 0.986 | <0.001 |  |
| Total Cholesterol | 31,168 | 1.000 | 0.001 | 0.9997, 1.001 | 0.536 |  |
| LDL | 27,126 | 0.995 | 0.001 | 0.994, 0.996 | <0.001 |  |
| High-density lipoprotein cholesterol | 25,257 | 0.982 | 0.002 | 0.979, 0.986 | <0.001 |  |
| Triglyceride | 28,078 | 1.000 | 0.001 | 1.000, 1.001 | <0.001 |  |
| Hemoglobin | 23,779 | 0.792 | 0.011 | 0.775, 0.809 | <0.001 |  |
| Uric acid | 11,673 | 1.283 | 0.012 | 1.255, 1.311 | <0.001 |  |
| SBP | 23,661 | 1.016 | 0.002 | 1.014, 1.019 | <0.001 |  |
| DBP | 23,662 | 0.986 | 0.003 | 0.981, 0.991 | <0.001 |  |
| Hypertension | 40,662 | 2.841 | 0.043 | 2.613, 3.089 | <0.001 |  |
| Hyperlipidemia | 40,662 | 1.314 | 0.037 | 1.224, 1.411 | <0.001 |  |
| CVD | 40,662 | 9.923 | 0.046 | 9.074, 10.85 | <0.001 |  |
| DR developed prior to CKD | 40,662 | 9.135 | 0.056 | 8.186, 10.19 | <0.001 |  |
| Biguanides use | 40,662 | 0.769 | 0.03 | 0.726, 0.815 | <0.001 |  |
| Sulphonylurea use | 40,662 | 1.698 | 0.034 | 1.592, 1.812 | <0.001 |  |
| Insulin use | 40,662 | 2.421 | 0.045 | 2.219, 2.641 | <0.001 |  |
| Alpha-glucosidase inhibitors use | 40,662 | 0.349 | 0.115 | 0.279, 0.437 | <0.001 |  |
| Dipeptidyl peptidase-4 inhibitors use | 40,662 | 1.867 | 0.048 | 1.700, 2.050 | <0.001 |  |
| Glucagon-like peptide-1 agonists use | 40,662 | 0.655 | 0.379 | 0.312, 1.375 | 0.263 |  |
| Thiazolidinediones use | 40,662 | 1.437 | 0.057 | 1.286, 1.605 | <0.001 |  |
| Sodium-glucose cotransporter-2 use | 40,662 | 1.102 | 0.231 | 0.702, 1.730 | 0.673 |  |
| Meglitinides use | 40,662 | 2.159 | 0.317 | 1.161, 4.015 | 0.015 |  |
| Statins use | 40,662 | 1.244 | 0.03 | 1.173, 1.319 | <0.001 |  |

*CV: Coefficient of variation; FPG: Fasting plasma glucose HbA1c: Haemoglobin A1c; SD: standard deviation*

Supplement table 5: Random Survival Forest Permutation Importance Top 20 Ranking

| **Cardiovascular disease (CVD)** | | | | |
| --- | --- | --- | --- | --- |
| **Feature Rank** | **HbA1c-CV model** | **HbA1c-SD model** | **FPG-CV model** | **FPG-SD model** |
| **1** | No antihypertensive drugs | No antihypertensive drug | No antihypertensive drug | Age |
| **2** | Age | Age | Age | No antihypertensive drug |
| **3** | Total Cholesterol | Total Cholesterol | CKD | CKD |
| **4** | CKD | CKD | Male | Total Cholesterol |
| **5** | Male | Male | Total Cholesterol | Male |
| **6** | Hypertension | DR | DR | Hypertension |
| **7** | DR | Hypertension | HDL | DR |
| **8** | HDL | HDL | Hypertension | HDL |
| **9** | DBP | DBP | DBP | DBP |
| **10** | BMI | BMI | Insurance scheme: National health insurance | LDL |
| **11** | Insurance scheme: National health insurance | Insurance scheme: National health insurance | LDL | FPG-SD |
| **12** | Hemoglobin | LDL | FPG-CV | Hemoglobin |
| **13** | Statins use | Sodium-glucose cotransporter-2 use | Statins use | BMI |
| **14** | SBP | Hemoglobin | SBP | Insurance scheme: National health insurance |
| **15** | Insulin use | SBP | Sodium-glucose cotransporter-2 use | Statins use |
| **16** | Sodium-glucose cotransporter-2 use | Statins use | Insurance scheme: Others | Sodium-glucose cotransporter-2 use |
| **17** | HbA1c-CV | HbA1c-SD | Biguanides use | Alpha-glucosidase inhibitors use |
| **18** | Biguanides use | Insulin use | Alpha-glucosidase inhibitors use | Insurance scheme: Social security insurance |
| **19** | LDL | Biguanides use | Glucagon-like peptide-1 agonists use | Thiazolidinediones use |
| **20** | Sulphonylurea use | Sulphonylurea use | Hyperlipidemia | Insurance scheme: Others |
| **Diabetes retinopathy (DR)** | | | | |
| **Feature Rank** | **HbA1c-CV model** | **HbA1c-SD model** | **FPG-CV model** | **FPG-SD model** |
| **1** | HbA1c-CV | HbA1c-SD | FPG-CV | FPG-SD |
| **2** | Insulin use | Insulin use | Insulin use | Insulin use |
| **3** | CKD | CKD | CKD | CVD |
| **4** | CVD | CVD | CVD | CKD |
| **5** | Insurance scheme: National health insurance | Insurance scheme: National health insurance | Sulphonylurea use | Sulphonylurea use |
| **6** | Sulphonylurea use | Sulphonylurea use | Insurance scheme: National health insurance | Insurance scheme: National health insurance |
| **7** | Thiazolidinediones use | Thiazolidinediones use | Thiazolidinediones use | Thiazolidinediones use |
| **8** | BMI | Biguanides use | BMI | BMI |
| **9** | Biguanides use | Hemoglobin | Age | Biguanides use |
| **10** | Age | BMI | SBP | SBP |
| **11** | Hemoglobin | HDL | Insurance scheme: Others | Insurance scheme: Others |
| **12** | HDL | Age | Biguanides use | Age |
| **13** | Male | Insurance scheme: Social security insurance | Male | Male |
| **14** | Statins use | Male | DBP | Hyperlipidemia |
| **15** | Glucagon-like peptide-1 agonists use | Sodium-glucose cotransporter-2 use | Total Cholesterol | Triglyceride |
| **16** | Meglitinides use | Glucagon-like peptide-1 agonists use | Triglyceride | Insurance scheme: Social security insurance |
| **17** | Sodium-glucose cotransporter-2 use | Alpha-glucosidase inhibitors use | Hyperlipidemia | Sodium-glucose cotransporter-2 use |
| **18** | Alpha-glucosidase inhibitors use | Meglitinides use | HDL | Meglitinides use |
| **19** | Insurance scheme: Social security insurance | Statins use | Hemoglobin | Glucagon-like peptide-1 agonists use |
| **20** | Total Cholesterol | DBP | Insurance scheme: Social security insurance | DBP |
| **Chronic kidney disease (CKD)** | | | | |
| **Feature Rank** | **HbA1c-CV model** | **HbA1c-SD model** | **FPG-CV model** | **FPG-SD model** |
| **1** | Age | Age | Hemoglobin | Hemoglobin |
| **2** | Hemoglobin | Hemoglobin | Age | Age |
| **3** | Male | CVD | CVD | FPG-SD |
| **4** | CVD | Male | DR | CVD |
| **5** | DR | DR | Male | DR |
| **6** | SBP | SBP | FPG-CV | Male |
| **7** | DBP | HbA1c-SD | Hypertension | SBP |
| **8** | HbA1c-CV | DBP | SBP | Hypertension |
| **9** | Hypertension | Hypertension | Sulphonylurea use | Insulin use |
| **10** | Insulin use | HDL | Insulin use | Total Cholesterol |
| **11** | Sulphonylurea use | Insulin use | BMI | Insurance scheme: National health insurance |
| **12** | Insurance scheme: National health insurance | Insurance scheme: National health insurance | DBP | HDL |
| **13** | Total Cholesterol | Sulphonylurea use | Triglyceride | BMI |
| **14** | Insurance scheme: Others | Total Cholesterol | Statins use | Sulphonylurea use |
| **15** | Insurance scheme: Social security insurance | Triglyceride | Sodium-glucose cotransporter-2 use | Biguanides use |
| **16** | HDL | Insurance scheme: Social security insurance | Glucagon-like peptide-1 agonists use | DBP |
| **17** | Meglitinides use | Glucagon-like peptide-1 agonists use | Meglitinides use | Sodium-glucose cotransporter-2 use |
| **18** | Glucagon-like peptide-1 agonists use | Statins use | Alpha-glucosidase inhibitors use | Thiazolidinediones use |
| **19** | Alpha-glucosidase inhibitors use | Meglitinides use | Hyperlipidemia | Meglitinides use |
| **20** | Sodium-glucose cotransporter-2 use | Alpha-glucosidase inhibitors use | Biguanides use | Hyperlipidemia |

Supplement Table 6: Left-truncated, right-censored (LTRC) Forest results

| **Cardiovascular disease (CVD)** | | | | | | | |
| --- | --- | --- | --- | --- | --- | --- | --- |
| **HbA1c (Tuned mtry = 2)** | | | | | | | |
| **No. of trees** | **Node depth** | **Integrated Brier Score**  **(Train)** | **Time taken** | **Integrated Brier Score**  **(Test)** | **Time**  **taken** | **C-index (Train)** | **C-index (Test)** |
| 16 | 6 | 0.0615 | 1.12 hr | 0.0645 | 29 min | 0.534 | 0.468 |
| 16 | 8 | 0.0605 | 1.04 hr | 0.0637 | 26 min | 0.558 | 0.474 |
| 16 | 9 | 0.0599 | 56 min | 0.0649 | 24 min | 0.596 | 0.492 |
| 16 | 10 | 0.0584 | 53 min | 0.0638 | 23 min | 0.637 | 0.497 |
| 32 | 6 | 0.0614 | 2.2 hr | 0.0642 | 57 min | 0.532 | 0.471 |
| 32 | 8 | 0.0598 | 2.01 hr | 0.0636 | 52 min | 0.574 | 0.489 |
| 32 | 9 | 0.0594 | 1.9 hr | 0.0640 | 49 min | 0.606 | 0.486 |
| 32 | 10 | 0.0582 | 1.8 hr | 0.0639 | 46 min | 0.638 | 0.499 |
| 64 | 6 | 0.0616 | 4.3 hr | 0.0649 | 1.9 hr | 0.532 | 0.460 |
| 64 | 8 | 0.0597 | 3.9 hr | 0.0635 | 1.6 hr | 0.593 | 0.484 |
| 64 | 9 | 0.0591 | 3.7 hr | 0.0632 | 1.6 hr | 0.610 | 0.497 |
| 64 | 10 | 0.0584 | 3.5 hr | 0.0635 | 1.5 hr | 0.648 | 0.503 |
| **FPG (Tuned mtry = 4)** | | | | | | | |
| **No. of trees** | **Node depth** | **Integrated Brier Score**  **(Train)** | **Time taken** | **Integrated Brier Score**  **(Test)** | **Time**  **taken** | **C-index (Train)** | **C-index (Test)** |
| 16 | 6 | 0.0606 | 1.3 hr | 0.0642 | 31 min | 0.564 | 0.484 |
| 16 | 8 | 0.0590 | 1.2 hr | 0.0645 | 29 min | 0.609 | 0.502 |
| 16 | 9 | 0.0574 | 1.1 hr | 0.0626 | 27 min | 0.654 | 0.527 |
| 16 | 10 | 0.0571 | 59 min | 0.0633 | 25 min | 0.674 | 0.531 |
| 32 | 6 | 0.0602 | 2.6 hr | 0.0632 | 1.08 hr | 0.557 | 0.484 |
| 32 | 8 | 0.0586 | 2.3 hr | 0.0628 | 58 min | 0.619 | 0.515 |
| 32 | 9 | 0.0580 | 2.2 hr | 0.0631 | 54 min | 0.639 | 0.517 |
| 32 | 10 | 0.0570 | 2 hr | 0.0626 | 51 min | 0.675 | 0.528 |
| 64 | 6 | 0.0600 | 5.0 hr | 0.0637 | 2.1 hr | 0.567 | 0.485 |
| 64 | 8 | 0.0586 | 4.5 hr | 0.0628 | 1.9 hr | 0.625 | 0.515 |
| 64 | 9 | 0.0578 | 4.2 hr | 0.0627 | 1.8 hr | 0.654 | 0.524 |
| 64 | 10 | 0.0571 | 4 hr | 0.0627 | 1.7 hr | 0.673 | 0.524 |
| **Diabetes retinopathy (DR)** | | | | | | | |
| **HbA1c (tuned mtry = 8)** | | | | | | | |
| **No. of trees** | **Node depth** | **Integrated Brier Score**  **(Train)** | **Time taken** | **Integrated Brier Score**  **(Test)** | **Time**  **taken** | **C-index (Train)** | **C-index (Test)** |
| 16 | 6 | 0.0303 | 1.2 hr | 0.0275 | 30 min | 0.734 | 0.549 |
| 16 | 8 | 0.0300 | 1.2 hr | 0.0276 | 30 min | 0.764 | 0.573 |
| 16 | 9 | 0.0296 | 1.1 hr | 0.0278 | 28 min | 0.805 | 0.594 |
| 16 | 10 | 0.0291 | 1.06 hr | 0.0274 | 27 min | 0.823 | 0.612 |
| 32 | 6 | 0.0303 | 2.3 hr | 0.0280 | 59 min | 0.731 | 0.569 |
| 32 | 8 | 0.0299 | 2.3 hr | 0.0276 | 57 min | 0.777 | 0.597 |
| 32 | 9 | 0.0293 | 2.2 hr | 0.0275 | 56 min | 0.801 | 0.602 |
| 32 | 10 | 0.0290 | 2.1 hr | 0.0274 | 54 min | 0.837 | 0.612 |
| 64 | 6 | 0.0303 | 4.7 hr | 0.0278 | 2 hr | 0.734 | 0.572 |
| 64 | 8 | 0.0297 | 4.5 hr | 0.0275 | 1.9 hr | 0.784 | 0.595 |
| 64 | 9 | 0.0294 | 4.4 hr | 0.0275 | 1.9 hr | 0.812 | 0.605 |
| 64 | 10 | 0.0290 | 4.2 hr | 0.0275 | 1.8 hr | 0.826 | 0.612 |
| **FPG (tuned mtry = 8)** | | | | | | | |
| **No. of trees** | **Node depth** | **Integrated Brier Score**  **(Train)** | **Time taken** | **Integrated Brier Score**  **(Test)** | **Time**  **taken** | **C-index (Train)** | **C-index (Test)** |
| 16 | 6 | 0.0288 | 1.4 hr | 0.0259 | 36 min | 0.694 | 0.620 |
| 16 | 8 | 0.0282 | 1.4 hr | 0.0257 | 34 mins | 0.749 | 0.660 |
| 16 | 9 | 0.0278 | 1.3 hr | 0.0256 | 32 mins | 0.792 | 0.666 |
| 16 | 10 | 0.0277 | 1.3 hr | 0.0257 | 32 mins | 0.792 | 0.651 |
| 32 | 6 | 0.0288 | 2.8 hr | 0.0261 | 1.2 hr | 0.701 | 0.636 |
| 32 | 8 | 0.0281 | 2.7 hr | 0.0256 | 1.1 hr | 0.768 | 0.656 |
| 32 | 9 | 0.0276 | 2.6 hr | 0.0257 | 1.1 hr | 0.794 | 0.676 |
| 32 | 10 | 0.0274 | 2.5 hr | 0.0255 | 1.0 hr | 0.812 | 0.678 |
| 64 | 6 | 0.0288 | 5.7 hr | 0.0260 | 2.4 hr | 0.712 | 0.634 |
| 64 | 8 | 0.0281 | 5.3 hr | 0.0256 | 2.2 hr | 0.763 | 0.662 |
| 64 | 9 | 0.0278 | 5.1 hr | 0.0255 | 2.1 hr | 0.794 | 0.675 |
| 64 | 10 | 0.0274 | 4.9 hr | 0.0256 | 2.1 hr | 0.823 | 0.678 |
| **Chronic kidney disease (CKD)** | | | | | | | |
| **HbA1c (tuned mtry = 4)** | | | | | | | |
| **No. of trees** | **Node depth** | **Integrated Brier Score**  **(Train)** | **Time taken** | **Integrated Brier Score**  **(Test)** | **Time**  **taken** | **C-index (Train)** | **C-index (Test)** |
| 16 | 6 | 0.0639 | 1.09 hr | 0.0623 | 27 min | 0.614 | 0.540 |
| 16 | 8 | 0.0625 | 59 mins | 0.0620 | 25 min | 0.651 | 0.561 |
| 16 | 9 | 0.0611 | 56 mins | 0.0621 | 24 min | 0.687 | 0.567 |
| 16 | 10 | 0.0602 | 51 min | 0.0618 | 22 min | 0.719 | 0.568 |
| 32 | 6 | 0.0635 | 2.1 hr | 0.0625 | 54 min | 0.602 | 0.532 |
| 32 | 8 | 0.0618 | 1.9 hr | 0.0624 | 48 min | 0.660 | 0.545 |
| 32 | 9 | 0.0608 | 1.8 hr | 0.0613 | 46 min | 0.695 | 0.566 |
| 32 | 10 | 0.0599 | 1.7 hr | 0.0613 | 44 min | 0.717 | 0.579 |
| 64 | 6 | 0.0637 | 4.4 hr | 0.0627 | 1.8 hr | 0.610 | 0.537 |
| 64 | 8 | 0.0615 | 1.6 hr | 0.0617 | 34 mins | 0.665 | 0.556 |
| 64 | 9 | 0.0608 | 3.7 hr | 0.0616 | 1.6 hr | 0.698 | 0.565 |
| 64 | 10 | 0.0599 | 3.56 hr | 0.0614 | 1.5 hr | 0.722 | 0.575 |
| **FPG (tuned mtry = 8)** | | | | | | | |
| **No. of trees** | **Node depth** | **Integrated Brier Score**  **(Train)** | **Time taken** | **Integrated Brier Score**  **(Test)** | **Time**  **taken** | **C-index (Train)** | **C-index (Test)** |
| 16 | 6 | 0.0611 | 1.2 hr | 0.0687 | 32 mins | 0.597 | 0.534 |
| 16 | 8 | 0.0585 | 1.1 hr | 0.0683 | 29 min | 0.661 | 0.556 |
| 16 | 9 | 0.0581 | 1.1 hr | 0.0681 | 27 min | 0.686 | 0.573 |
| 16 | 10 | 0.0573 | 1 hr | 0.0684 | 25 min | 0.705 | 0.555 |
| 32 | 6 | 0.0605 | 2.4 hr | 0.0687 | 1 hour | 0.606 | 0.530 |
| 32 | 8 | 0.0590 | 2.2 hr | 0.0690 | 55 mins | 0.654 | 0.544 |
| 32 | 9 | 0.0579 | 2.1 hr | 0.0686 | 52 mins | 0.696 | 0.550 |
| 32 | 10 | 0.0572 | 2.0 hr | 0.0683 | 51 mins | 0.712 | 0.561 |
| 64 | 6 | 0.0604 | 4.8 hr | 0.0685 | 2.0 hr | 0.608 | 0.542 |
| 64 | 8 | 0.0585 | 4.3 hr | 0.0681 | 1.8 hr | 0.669 | 0.555 |
| 64 | 9 | 0.0576 | 4.1 hr | 0.0677 | 1.8 hr | 0.692 | 0.564 |
| 64 | 10 | 0.0569 | 4.2 hr | 0.0679 | 1.7 hr | 0.718 | 0.572 |
